# Neuroprotective efficacy and therapeutic window of curcuma oil: in rat embolic stroke model

**DOI:** 10.1186/1472-6882-8-55

**Published:** 2008-09-30

**Authors:** Preeti Dohare, Puja Garg, Uma sharma, NR Jagannathan, Madhur Ray

**Affiliations:** 1 Division of Pharmacology, Central Drug Research Institute, P.O Box no 173, Chattar Manzil Palace, Lucknow, U.P, 226001, India; 2 All India Institute of Medical Sciences, New Delhi, India

## Abstract

**Background:**

Among the naturally occurring compounds, turmeric from the dried rhizome of the plant *Curcuma longa *has long been used extensively as a condiment and a household remedy all over Southeast Asia. Turmeric contains essential oil, yellow pigments (curcuminoids), starch and oleoresin. The present study was designed for investigating the neuroprotective efficacy and the time window for effective therapeutic use of Curcuma oil (C. oil).

**Method:**

In the present study, the effect of post ischemic treatment of C.oil after ischemia induced by occlusion of the middle cerebral artery in the rat was observed. C.oil (500 mg/kg body wt) was given 4 hrs post ischemia. The significant effect on lesion size as visualized by using diffusion-weighted magnetic resonance imaging and neuroscore was still evident when treatment was started 4 hours after insult. Animals were assessed for behavioral deficit scores after 5 and 24 hours of ischemia. Subsequently, the rats were sacrificed for evaluation of infarct and edema volumes and other parameters.

**Results:**

C.oil ameliorated the ischemia induced neurological functional deficits and the infarct and edema volumes measured after 5 and 24 hrs of ischemia. After 24 hrs, immunohistochemical and Western blot analysis demonstrated that the expression of iNOS, cytochrome *c *and Bax/Bcl-2 were altered after the insult, and antagonized by treatment with C.oil. C.oil significantly reduced nitrosative stress, tended to correct the decreased mitochondrial membrane potential, and also affected caspase-3 activation finally apoptosis.

**Conclusion:**

Here we demonstrated that iNOS-derived NO produced during ischemic injury was crucial for the up-regulation of ischemic injury targets. C.oil down-regulates these targets this coincided with an increased survival rate of neurons.

## Background

*Curcuma longa Linn*, commonly known as turmeric, is a perennial plant belonging to the family Zingiberaceae. This common Indian dietary spice and pigment has had a prominent place in the armentarium in the traditional Indian medicine. The active constituents of turmeric are categorized into the non-volatile components curcumin and other curcuminoids and the volatile curcuma oil. The content of the curcuma oil are the turmerones and other sesquiterpenoid cyclic ketones [1]. The highly lipophilic nature of the Curcuma oil may have as it is reported to have access to the brain after stroke through the transcellular lipophilic pathway. C.oil has been reported in scientific literature for antimicrobial, antifungal, antiviral [2], anti-inflammatory activities, wound healing, insecticidal activity [3] and, of late, for its potent effect in oral sub-mucosal fibrosis in human [4].

The chemical components in oil extracted from the rhizome of *Curcuma longa *after identification of the chemical constituents with the help of GC, GC-MS are recently reported [5]. The standardization of C.oil was carried out by using HPLC. Sesquiterpenoids are the major constituents of turmeric oil and ar-turmerone is the major turmerone, identified by GC-MS analysis. We have recently reported the beneficial antioxidant effects of pretreatment by C.oil in the filament model of middle cerebral artery occlusion rat [6], this model of focal ischemia has been associated with generation of free radicals [7]. Cerebral ischemia is associated with enhanced oxidative and nitrosative stress. A substantial proportion of cell death after brain ischemia results directly or indirectly from oxidative/nitrosative injury. In our another recent report on neuroprotective effect of the pretreatment of C.oil in filament model of middle cerebral artery occlusion, is due to attenuation of oxidative/nitrosative stress [8].

In the clinical scenario, the time point at which medical intervention becomes available is very critical. Most of the studies in the past were carried out by pretreatment of test agents. This is not a promising approach in the clinical scenario. This is evident from the finding that in the first hour of reperfusion after two hours of occlusion of the middle cerebral artery (MCAo), the bioenergetics state of the tissue is extensively restored, but secondary deterioration is observed after 4 hrs of recovery and thereafter [9]. Expression of inducible nitric oxide synthase (iNOS) occurs in the later stage of cerebral ischemia, and NO produced by iNOS contributes to delays in recovery from brain neuronal damage.

While there are many reasons why experimental neuroprotection has been difficult to replicate in patients, one major area is appropriateness of the animal models used. It has been suggested that the embolic stroke model encompassing a more 'physiological' spontaneous reperfusion process, more akin to the clinical pathology, and with a low mortality rate and excellent reproducibility [10] would be more predictive. The other reason for using the embolic stroke model is on the basis of recommendations of the "Stroke Therapy Academic Industry Roundtable" which have specifically recommended particularly that preclinical neuroprotective activity for cerebral ischemia should be tested in multiple cerebral ischemia models in order to get the ideal neuroprotective agent [11,12].

Another important point of surviving stroke patients is disability and dependence because of functional impairment. In clinical studies, functional outcome after an ischemic episode is one of the most useful parameters. We associated in the present study evaluation of functional outcome (neurological deficit score) with lesion size along with progression of infarct. For this region all parameters were studies after 24 hrs of ischemia when significant neurological deficits were observed. In this study, we explore the effects of post-ischemic treatment by C.oil in an embolic stroke model in the rat for its neuroprotective efficacy and therapeutic window. Prior to the present study a good correlation between concentration of major constituents by HPLC and GC of C.oil was done [5].

## Methods

### Experimental Procedure

#### Animals

Male Sprague Dawley rats (maintained at the Animal House facility of the Central Drug Research Institute at Lucknow) were used in the present study. All animal experiments were carried out in strict accordance with the Institute guidelines on the care and use of experimental animals. Rats were fasted overnight (12 hrs–16 hrs) with free access to water before surgery.

#### Extraction of C.oil

Curcuma oil isolation: After botanical identification sample of the dried rhizomes of *Curcuma longa *of Zingiberaceae family were preserved by the Botany department of the Central Drug Research Institute, India, for future reference. Rhizomes were powered and extracted at room temperature with light petroleum ether. The extract was concentrated at low temperature to remove petroleum ether; the oily residue was collected and dried. The identification of chemical constituents present in C.oil was done with GC, GC-MS. The standardization of C.oil was done using HPLC method and a mixture of a group of terpenoids were isolated and identified. The chemical profile of curcuma oil was done with GC-MS. It is a pale yellow to orange yellow odoriferous oil. Recently Raina et al., (2002) reported that curcuma oil comprised of 100% of oil and contained 84 constituents [13].

**Figure 1:**
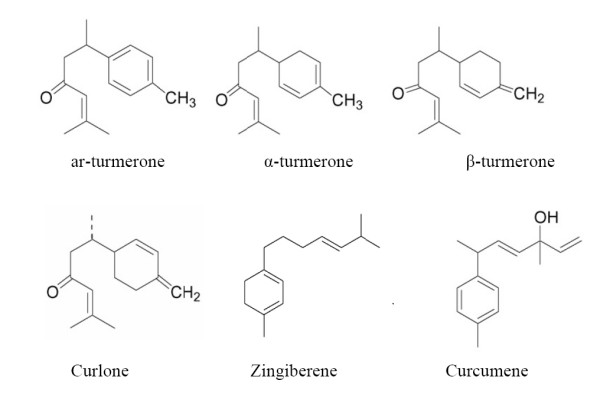
The chemical structures of the major constituents obtained from Curcuma oil ar-turmerone; turmerone; curlone; Zingiberene and Curcumene.

Major constituents of curcuma oil were ar-turmerone, α-β turmerone, and curlone (Fig. 1). Four major curcumenes: curcumene, zingiberene, bisabolene and sesquiphellandrene and other minor constituents like zederone, germacrone, curdione, curcumerone, zederondiol and isozederendiol and three curcuminoids: mono-demethoxy curcumin, di-demethoxy curcumin, curcumin were identified. The stability with change in pH and other physiochemical properties like photosensitivity etc has been published recently [5,14]. C.oil was given 500 mg/kg and three marker compounds ar-turmerone, α-β turmerone and curlone were selected for plasma concentration time profile. The C_max _(ng/ml) for ar-turmerone turmerones, α-β turmerone and curlone was 412 ± 97.4, 316 ± 129.7 and 21.4 ± 6.6 respectively and T_max _was 2 hrs (unpublished observation).

### Experimental groups

All the animal were neurologically evaluated before the start of experiments and any rat showing any deficit were not included in the study.

Following groups of five rats were assigned. (A) Sham-operated rat euthanized at 5 and 24 hrs for quantification of edema and infarct volumes and other parameters; (B) peanut oil treatment 4 hrs after ischemia and evaluation was done 5 hr for Diffusion-Weighted Magnetic Resonance Imaging (DW-MRI). (C) Peanut oil or curcuma oil treatment was given at 4 hrs after ischemia. After 1 hr of treatment evaluation was done for neurological scores. Thereafter rats were euthanized for quantification of edema and infarct volumes (D) Peanut oil or Curcuma oil was given at 4 hrs after ischemia. After 6, 12 and 24 hrs of ischemia brain tissue nitrate/nitrite levels were estimated. For sham-operated no treatment was given. (E) Peanut oil or Curcuma oil was given 4 hrs after ischemia and neurological evaluation were made after 24 hrs of ischemia. Thereafter rats were euthanized for quantification of edema and infarct volumes and other parameters.

To run a parallel control, rats from ischemic group were given commercially available peanut oil in equal volume. In our preliminary experiments, peanut oil and normal saline were used as control treatments and the behavioral data did not differ between the rats that received the two vehicle solutions [15]. Therefore, we chose to present only the peanut oil control group data for comparison.

The dose of C.oil was chosen according to the pharmacokinetic properties of C.oil in experimental animals (data not shown) as well as our dose response experiments. The C.oil 500 mg/kg was administered after 4 hrs of clot implant via the oral route as such to rats and no vehicle was used in the study. The reason for oral route delivery was based on the earlier published report of [5] that the oil was most effective in the treatment of bronchial asthma by oral route than when given intramuscularly.

To rule out the possibility of spontaneous re-canalization before C.oil treatment, whether the attenuation that was observed in the C.oil treated animals was due to the treatment, and not because of the reperfusion effect prior to treatment, it was very important to demonstrate that all animals had equivalent ischemic state (i.e. before treatment). For this reason Diffusion-Weighted Magnetic Resonance Imaging (DW-MRI) recordings were taken from each rat before and after ischemia and again after C.oil treatment in treated rats. The same protocol was followed for the peanut oil treated ischemic and sham-operated rats. All the untreated rats were firstly scanned for DW-MRI and any rat showing any significant lesion was excluded from the study. The same rats were evaluated for neurological deficit after 24 hrs of ischemia. Thereafter these rats were euthanized and post mortem brain infarcts and edema volumes were determined. For the rest of experiments, separate group of rats were used and evaluations were made 24 hrs after ischemia.

#### Focal Embolic Model

To induce reproducible lesions obstructions by emboli should be in the proximal segment of a large feeder artery [16]. Embolic cerebral ischemia was induced as reported earlier by injecting multiple fibrin-rich autologous clots one after another into the external carotid artery [16,17]. For clot formation blood was drawn from the femoral vein of a donor rat and left to form a clot as reported [16]. The clot was fragmented by extruding it twice through a 27-gauge needle and washed. Multiple fibrin-rich autologous clots formed in this way.

Briefly, for producing embolic cerebral ischemia rats were anaesthetized with chloral hydrate (400 mg/kg, i.p.). The right common carotid artery (CCA), the right internal carotid artery (ICA) and the right external carotid artery (ECA) were exposed by a ventral incision in throat. The multiple clots suspended in phosphate with phosphate-buffered saline (0.3 ml) were taken up in a Hamilton syringe connected to a PE 50 catheter. The tip of the catheter was advanced up to 17 mm into the internal carotid artery via the stump of the external carotid artery and the clot were lodge into the MCA stem during which time the common carotid artery was temporarily closed. Selection of appropriate clot and by washing results to high fibrin content of clots ensures durability and delays the spontaneous lysis. After removal of the catheter, the clips on the right CCA and the right ECA were removed and the incision was closed. The sham-operated rats underwent same surgical procedure and a PE 50 catheter was advanced up to 17 mm in the ICA via ECA and immediately withdrawn without leaving behind a clot implant. The rectal temperature in the animals was kept at 37°C throughout the surgical procedure with the help of heating pad.

#### Early detection of lesion area using diffusion-weighted MRI (DW-MRI)

MRI was used to examine ischemic rat brain after 1 hr of C.oil treatment. The DW-MRI scan was taken from same rat, before, after ischemia and again about 1 hr after C.oil or peanut oil treatment. All DW-MRI experiments were carried out according to Rathore et al (2008)[6], using an animal MRI/MRI scanner (Bruker, BIOSPEC). A 69-mm circularly polarized birdcage volume resonator was used to carry out experiments at 4.7 T. The seven slices acquired were summed to provide the total volume of ischemic injury. The threshold value of 15% was found to be the lowest cutoff value that did not include pixels in the contra lateral non ischemic hemisphere [18]. Result of seven coronal sections each brain of five rats per group is given.

#### Neurological Evaluation

After 5 hrs and 24 hrs of DW-MRI evaluation the rats from sham-operated, peanut oil and C.oil post treated groups were evaluated for neurological deficit. An observer who was unaware of the identity of the groups assessed neurological deficits and gained scored as described by the [19,20] ; as follows: 0–no observable neurological deficit (normal); 1–failure to extend left forepaw on lifting the whole body by tail (mild); 2–circling to the contralateral side (moderate); 3–leaning to the contralateral side at rest or no spontaneous motor activity (severe). Result of neurological deficit evaluation were made from ten rats/group and expressed as median/range.

#### Determination of cerebral infarct and edema

The further study was planned to correlate the lesion assessed in vivo by DW-MRI and infarct and edema volumes. The rational of this study was that the change in lesion volume at the early stage of stroke on total evolution of thromboembolic stroke, because recent therapy in acute stroke is based on reperfusion of the ischemic legion leading to salvage of the ischemic penumbra, smaller infarct size, and improved outcome [21].

Rats from all the groups that were initially evaluated for DW-MRI at 5 hrs and 24 hrs and thereafter evaluated for neurological deficits. The rats were euthanized and the brains were quickly removed and cooled on ice for a few min and then coronally sectioned by a tissue slicer in 2-mm thick sections starting from 2 mm caudal to the frontal tip. Each slice was examined for subarachnoid hemorrhage. The slices were immediately stained with 1% 2, 3, 5-triphenyltetrazolium chloride (TTC) (Sigma) at 37°C for 15 min and fixed immersion in 4% phosphate-buffered formalin solution [20,22]. The infarct area and edema volume in seven coronal sections of each brain was determined using the scanned image with a CCD camera (Samsung, Korea) and quantified with Biovis, Expert vision, (India). The total infarct volume was determined by summing up the infarct areas of the seven sections which was multiplied by thickness of brain sections (2 mm) to get the volume of infarction and was corrected for edema volume. Edema correction of infarct volume was done using the equation, volume correction = (infarct volume × contra lateral volume)/ipsilateral volume. The volumes of both the contra lateral and the ipsilateral hemispheres were calculated from the product of the average slice thickness (2 mm) and the sum of areas in all seven brain slices, and edema volume was calculated by subtracting the contralateral volume from the ipsilateral volume.

#### Measurements of nitrate and nitrite levels

The final products of NO in vivo are nitrate and nitrite, and thus the sum of both species is an index of total NO production [23]. Nitrate and nitrite content was estimated at 6, 12 and 24 hrs after ischemia. Rats from sham-operated, peanut oil treated ischemic and C.oil treated rats were euthanized at 6, 12 and 24 hrs. Briefly, animals were euthanized and the brains were quickly removed. Ipsilateral sides of forebrain from each group were homogenized in 3 volumes (w/v) of PBS (pH 7.4) at 4°C. Homogenates were then sonicated and centrifuged at 100,000 *g *for 60 min at 4°C. The nitrate plus nitrite (NOx) values were determined in the supernatants using a colorimetric kit according to the manufacturer's instructions (Nitrate/Nitrite colorimetric assay kit, Cayman Chemical) by a simple two-step process. The first step was the conversion of nitrate to nitrite utilizing nitrate reductase and NADPH at room temperature for 3 hrs. The second step was the measurement of nitrite content by the Griess reaction, by adding 100 μl of Griess reagent to 100 μl of samples. Griess reagent converts nitrite into a deep purple azo compound. Photometric measurement of the absorbance at 550 nm due to this azo chromophore using ELISA micro plate reader (Power wave XS, BIO-TEK, U.S.A.) accurately determines NO_2_^- ^concentration. Nitrite concentration was calculated by comparison with OD_550 _of standard nitrite solution [24]. Results are expressed as μmol/mg protein.

### Myeloperoxidase estimation

Myeloperoxidase (MPO) activity, an indicator of polymorphonuclear leukocyte (PMN) accumulation, was determined as previously described [25]. Rats from sham-operated, peanut oil treated ischemic and C.oil treated rats were euthanized after 24 hrs of ischemia and MPO activity was estimated. Briefly, ipsilateral side of forebrain were homogenized (Polytron, three times at 5-second bursts) in 20 times the volume of 5 mmol/L phosphate buffer (pH 6.0) at 4°C, followed by centrifugation at 30,000 *g *for 30 minutes at 4°C. The supernatant was discarded, and the pellets were extracted by suspending in 10 times the volume of 0.5% hexadecyl trimethyl ammonium bromide in 50 mmol/L phosphate buffer (pH 6.0) at 25°C. These samples then were frozen on ice, with three freeze/thaw cycles, and sonicated for 10 seconds at 25°C. After sonication, the samples were incubated for 20 minutes at 4°C and centrifuged at 12,500 *g *for 15 minutes at 4°C. MPO activity in the supernatant was assayed reported earlier by us [22].

### Tissue caspase-3 and caspase-1& 4 estimation

The fluorometric substrate Ac-DEVD-AMC and Ac-YVAD-AMC were used for the determination of caspase-3 and caspase-1&4 activities. Caspase 1&4 estimation was made 24 hrs after ischemia. Rats from sham-operated, peanut oil treated ischemic and C.oil treated rats were euthanized after 24 hrs. Ipsilateral side of forebrains were quickly isolated, washed with phosphate buffered saline and cells lysed in 20 mM Tris HCl buffer (pH 7.4) containing 150 mM NaCl, 1 mM dTT, 5 mM EDTA, 5 mM EGTA and 1% w/v Triton X-100. Tissue lysate was obtained by centrifugation at 100,000 *g *for 30 min. Cleared lysate containing 50–100 μg of protein was assayed with 100 μM of the enzyme substrate Ac-DEVD-AMC and Ac-YVAD-AMC in assay buffer (100 mM Hepes, 10% glycerol, 1 mM EDTA, 10 mM dTT) and incubated for 60 minutes at 37°C. Absorbance of the cleaved product was measured using a Fluorimeter (Varian Cary Eclipse) with excitation at 380 nm and emission at 460 nm and the activity was expressed as percent of substrate released over control.

### Flowcytometric estimation of nitric oxide (NO), reactive oxygen species (ROS) intracellular calcium level (Ca^++^), peroxynitrite (ONOO^-^), and mitochondrial membrane potential (ΔΨm)

Estimations were done after 24 hrs of ischemia. Rats from sham-operated, peanut oil treated ischemic and C.oil treated rats were euthanized after 24 hrs and brains were quickly removed. For neuron isolation the forebrain of ipsilateral side was dissected and processed according to the method detailed by Rathore et al (2008), Dohare et al (2008) [6,20] and Rastogi et al (2006) [22]. In neuronal rich cell population were used for the estimation of ROS, NO, ONOO^-^, mitochondrial membrane potential (ΔΨm), intracellular calcium level (Ca^++^) and quantification of cell death type. A homogenous suspension of individual neurons was prepared in PBS, (pH 7.4) by treatment of finely chopped forebrain tissue with dispase (1000 protease unit/ml) for 45 min at 37°C. After enzymatic treatment, the dissociated neurons were passed through a filter (~53 μm) to remove large neurons and tissue fragments as reported by us [6,20]. Confirmation of neuron population was done by labeling the neurons with NeuN (a monoclonal antibody against neuron-specific marker NeuN conjugated with PE; SC7301, Santacruz; dilution 1:50). NeuN positive neurons were gated in the dot plot and the same gating was used in the entire study. Fluorescence was determined in a flowcytometer (Becton Dickinson, UK, Ltd). Data were analyzed using Cell Quest Software (Becton Dickinson UK, Ltd). For each sample, 10,000 neurons (events) were acquired. The flowcytometry data was expressed as mean ± S.E.M. from five animals per group of each rat brain.

#### Nitric oxide measurement

For NO estimation, isolated neurons (10^6^/ml) were incubated with Diaminofluorescein diacetate (DAF-2DA) (10 μM) for 30 min at 37°C. Fluorescence was measured at an excitation wavelength of 495 nm and emission wavelength of 515 nm [26].

#### Reactive oxygen species measurement

ROS was measured with the help of 2', 7'dichlorodihydrofluorescein diacetate (DCDHF-DA). Isolated neurons (10^6^/ml) were incubated with 50 μM of DCDHF-DA for 30 min at 37°C. Fluorescence was measured at an excitation wavelength of 488 nm and emission wavelength of 530 nm [6].

#### Intracellular calcium measurement

For measurements of intracellular calcium the neurons were washed twice with PBS without calcium and magnesium. After washing, the cells were re-suspended at 2 × 10^6 ^cells/ml in PBS and incubated for 30 min at 37°C with 100 μg/ml Pluronic F-127 and 7.5 μM of the fluorescent calcium probe Fluo-3AM. After incubation, the cells were washed twice with PBS. Fluorescence was measured at excitation wavelength of 506 nm and emission wavelength of 526 nm [27].

#### Peroxynitrite measurement

The direct reaction of DHR 123 with H_2_O_2 _does not occur but can be catalyzed by heme-containing peroxidases. Neurons were loaded with DHR 123 and it was necessary to check the genuineness of the signals obtained. SOD diminishes the signals due to peroxynitrie while CAT which reacts with H_2_O_2 _should not. We did the pilot experiments and have reported in our recent paper [8]. The formation of ONOO^- ^was measured by the ONOO^- ^dependent oxidation of dihydrorhodamine-123 (DHR-123). Isolated neurons (10^6^/ml) were incubated with 5 μM DHR-123 for 60 minutes at 37°C. After incubation, 123-DHR conversion to 123-rhodamine was measured at excitation wavelengths of 500 nm and emission wavelength of 536 nm [28].

#### Mitochondrial membrane potential (ΔΨm) measurement

The mitochondrial membrane potential (ΔΨm) was measured by the retention of Rhodamine 123, a cationic dye, which accumulates electrophoretically into energized mitochondria in response to their negative inside membrane potential. Neurons (10^6^/ml) were loaded with Rhd 123 (1.3 μM) for 30 min [29].

#### Neuronal death measurement

For quantification of mode of neuronal death neurons isolated were labeled with FITC (Fluorescene Iso thio-cyanate) conjugated Annexin V and Propidium iodide (PI). Loading of fluorochromes was done according to the manufacturer's protocol (Calbiochem, Apoptosis Detection kit). In brief, after incubation at room temperature for 15 min in the dark, the cells were analyzed by FACScan analyzer at an excitation wavelength of 488 nm and emission wavelength of 530 and 670 nm. When the two fluorochromes were used simultaneously, the dead PI positive cells fall in the upper left quadrant, the apoptotic Annexin-V FITC positive cells in the lower right quadrant, viable neurons negative for both of these stains fall in the lower left quadrant. Late apoptotic/necrotic which were positive for both PI and Annexin-V FITC fall in upper right quadrant [6,8,22].

### Immunohistochemical analysis of iNOS, eNOS and cytochrome c expression

Expression of iNOS, eNOS and cytochrome *c *were estimated 24 hrs after ischemia in brain cryosections from each brain of five rats/group. Rats from sham-operated, peanut oil treated ischemic and C.oil treated rats were euthanized 24 hrs after ischemia. Expression of iNOS, eNOS and cytochrome *c *were detected in the cryosections by immunohistochemical analysis using highly specific antibodies [30]. In brief, brains were placed on frozen section medium Neg50 (Richard-Allan Scientific) for cryostat sections. Six micrometer-thick sections were cut by a cryostat (Microm HM 550) and mounted on slides coated with poly L-lysine (0.1% w/v in water). A maximum of five slides per brain, with several sections on each slide, in a pre-selected region of interest were taken. Brain sections at roughly the same coronal level, approximately 2.8 mm posterior to the bregma, containing the dorsal hippocampus, corpus callosum and thalamus were chosen. These brain tissue sections were first fixed in 4% paraformaldehyde for 1 hr and then after washing in 0.1% glycine PBS (Phosphate buffered saline, pH 7.4) were used for quenching. Sections were than permeabilized with 0.1% Triton X-100 for 10 minutes. Sections were washed with PBS and blocked in blocking buffer (0.5%BSA in PBS) for 1 hr to reduce non-specific staining. Sections were incubated overnight with primary polyclonal iNOS antibody conjugated with FITC (SC-651; Santacruz, 1:200) primary antibody for eNOS (SC-653; Santacruz, 1:200) and cytochrome *c *(SC-7159; Santacruz, 1:200), diluted in blocking buffer to 1:200 ratio and then rinsed 5 times for 5 min each time in PBS. After washing, sections were treated with DAPI (300 nM) for counterstaining for 5 min at room temperature and again washed 5 times for 5 min and then the sections were incubated with secondary anti-rabbit polyclonal antibody conjugated with FITC (Santa Cruz Biotechnology, USA), diluted in blocking buffer to 1:400 ratio for 4 hrs. After washing, sections were mounted in 90% glycerol with anti-fading agent (PPD). All the sections were analyzed using IM 50 software in a Leica D2LB microscope and images from the same cortical region were captured with the associated software. Images were further processed using Photoshop software (Adobe, San Jose, CA). For negative control only the primary antibodies were omitted.

### Expression of iNOS, eNOS, cytochrome c, Bax and Bcl-2 by Western Blot

Western blot analysis of iNOS, eNOS, cytochrome *c*, Bax and Bcl-2 expression in the forebrains of the rats was carried out after 24 hrs of ischemia. Rats from sham-operated, peanut oil treated ischemic and C.oil treated groups were euthanized after 24 hrs of ischemia. Five rats/group were used for the study. Ipsilateral side of forebrain was dissected and homogenized at 4°C in a Tris-HCL buffer (50 mM, pH 7.6) containing: 150 mM KCL, 5 mM ethylenediamine tetraacetic acid (EDTA), 1 mM phenylmethane sulfonylfluoride (PMSF), 1% Triton X-100, 0.5 μg/mL leupeptin and 1 μg/mL pepstatin [30]. The homogenate was centrifuged at 15,000 g for 20 min and the supernatant used as the cytosolic fraction. Total cellular proteins (50 μg/lane cytosolic fractions) were separated on a 10% SDS-polyacrylamide gel and electro-transferred onto PVDF membranes (Hybond ECL, Amersham International, Bucks, U.K.). Equal loading and transfer of proteins was confirmed by temporally staining the membranes with Ponceau S solution. Nonspecific binding of proteins was prevented by treating the membranes with 5% nonfat dry milk in Tris-buffered saline/0.1% Tween 20 for 2 hrs at room temperature. The membranes were incubated overnight at 4°C with anti iNOS (1:1000; Santacruz), anti eNOS (1:1000; Santacruz), anti cytochrome *c *(1:500; Santacruz), anti-Bax (1:500; Oncogene) and anti-Bcl-2 antibody (1:500; Oncogene). After incubation with secondary antibody conjugated to horseradish peroxidase (anti-rabbit, 1:20000; Santacruz), immunoreactive proteins were detected by the enhanced chemiluminescence's system (ECL, Sigma) and serial exposures were made to radiographic film (Hyper film ECL, Amersham International).

### Protein estimation

Protein content of the supernatant was determined using bovine serum albumin (BSA fraction V, Sigma) as the protein standard by the method of Lowry et al (1951) [31].

### Chemicals/Reagents used

Ac-DEVD-AMC, Ac-YVAD-AMC, bovine serum albumin, dispase, dithiothreitol, 2', 7'dichlorodihydrofluorescein diacetate, dihydrorodamine-123, diaminofluorescein diacetate, ethylenediamine tetraacetic acid, ethyleneglycol-bis-(β-aminoethyl ether) N,N,N'N'-Tetraacetic acid, Fluo-3AM, Glycine, Glycerol, hexadecyl trimethyl ammonium bromide, leupeptin, O-Dianisidine-HCl, paraformaldehyde, pepstatin, phenylmethane sulfonylfluoride, Pluronic F-127, ponceau S, propidium iodide, sodium azide, sodium dodecyl sulphate, Tris-HCl, Triton X-100, tween 20, triphenyltetrazolium Chloride and enhanced chemiluminescence system were procured from Sigma Chemical Co., St. Louis MO, USA). PVDF membrane and Hyperfilm ECL were procured from Amersham International (Bucks, U.K.). Primary and secondary antibodies were purchased from Santacruz (California, USA) and Oncogene (CA, USA). Nitrate/Nitrite colorimetric measurement kit was from Alexis (CA, USA), and Apoptosis Detection kit was from Calbiochem (Germany). Other chemicals of AR grade were obtained from SRL and Qualigens, India.

### Statistical analysis

All values (except neurological deficits) in the figures are expressed as means ± standard error of the mean (S.E.M.). The results were analyzed by One-way analysis of variance (ANOVA) with post hoc comparison (Newman Keuls multiple) test as per nature of data. For neurological deficit scores, the nonparametric data were analyzed by Kruskal Wallis test followed by Dunn's multiple comparison test. P-value of less than 0.05 was considered significant. All the analyses were performed with Graph Pad prism software (Ver. 3.03).

## Results

### Lesion volume analysis by MRI studies

**Figure 2:**
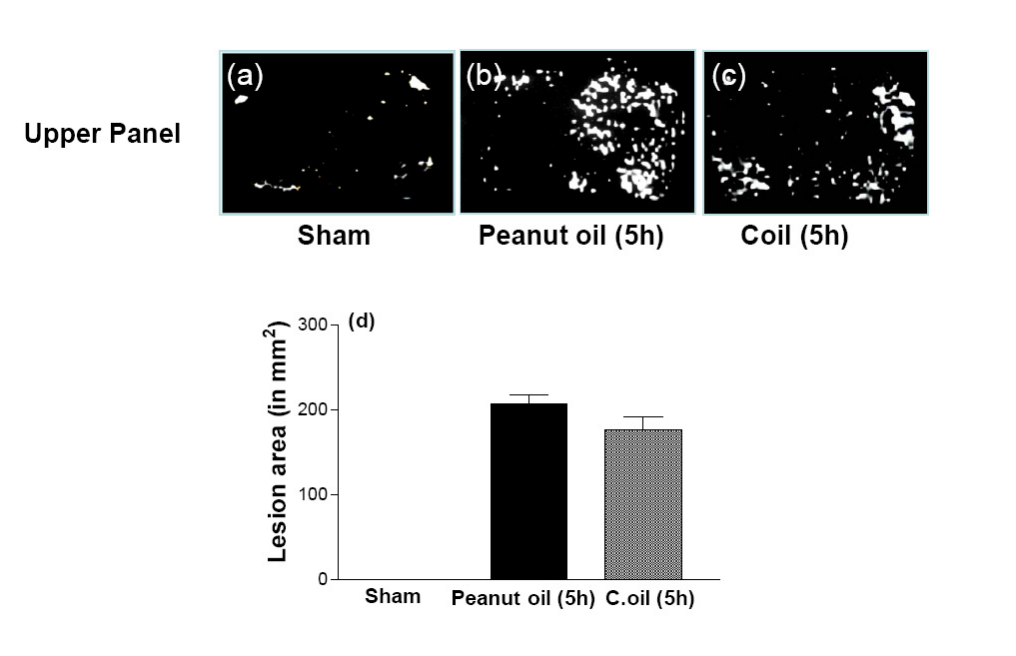
**Effect of C.oil post-treatment on lesion area of brain at 5 hrs.** The MRI scan was taken from same rat, before, after ischemia and 1 hr after peanut oil treatment- the ischemic group. In the second set of rats MRI scan was taken before and after ischemia and again after 1 hr of C.oil treatment. Hyper intense areas represent the lesion. Upper panel shows the representative photograph of brain scan from brains of sham-operated (a), 5 hrs after rat treated with peanut oil (b) and 5 hrs after rat treated with C.oil (c). Bar graph (d): The bar chart showing the lesion area of brains of sham-operated, peanut oil treated ischemic and C.oil post-treatment ischemic group at 5 hrs. Data are expressed as mean ± S.E.M. *P *< 0.001 was considered highly significant when comparisons were made with the peanut oil treated ischemic group by one way ANOVA followed by Newman Keuls post hoc test. Data are from five rats per group.

Diffusion-weighted Magnetic resonance Imaging (DWI) is very sensitive as it can detect ischemic lesions in the hyperacute phase of ischemia even within minutes of injury [32]. The greater the neuronal damage the brighter the DWI lesion, [33]. After 3 hrs of ischemia, rats showed significant increase in the lesion area in the ischemic hemisphere (207.4 ± 10.2 arbitrary units). The significant effect on lesion size and neuroscore was still evident when treatment of C.oil was started 4 hours after insult. The signal intensity in the ischemic hemisphere after C.oil treatment was 176.7 ± 14.78 as compared to its ischemic value (before the treatment). C.oil or peanut oil was given after 4 hrs of ischemia the animals were again scanned after 1 hour. In the peanut oil post treated group, the difference in the signal intensity of the ischemic hemisphere was not statistically significant as compared to its previous scans (Fig. 2 Upper panel and d). These observations rule out the possibility of spontaneous re-canalization before C.oil treatment. The DW-MRI data show that the neuroprotective effect of C.oil was observed as early as 1 hour post oral treatment with the oil. Since the animals treated with peanut oil not show any reduction in the signal intensity after treatment and the group treated with C.oil did show significant reduction in intensity, a reasonable conclusion would be that the effect seen in the C.oil treated group would be due to the C.oil and not due to spontaneous reperfusion.

### Neurological deficits

**Figure 3:**
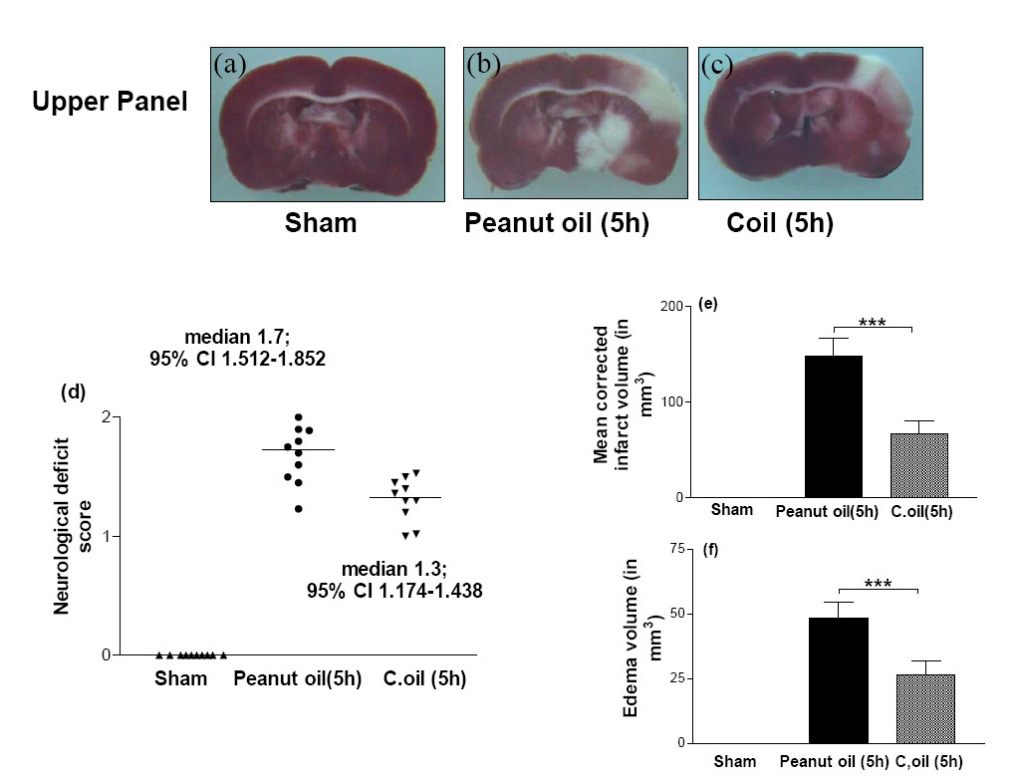
**Protective effect of C.oil on brain infarct and edema volumes and neurological evaluation from rat subjected to middle cerebral artery occlusion at 5 hrs.**Upper panel showing the representative photograph of TTC stained coronal brain section after 5 hrs of ischemia from sham-operated (a), peanut oil treated ischemic (b) and ischemia with C.oil post-treatment (c) group at 5 hrs. Unstained areas represent the infarcted brain tissue. Bar graph (d): Neurological evaluation scores of rats from sham-operated, peanut oil treated ischemic and C.oil post-treatment ischemic groups after 5 hrs of ischemia as shown in scattergram. The line demarcates the median. Comparisons were made between C.oil post treated vs. peanut oil treated ischemic group by nonparametric Kruskal-Wallis test followed by Dunn's Multiple Comparison Test. Ten rats/group was used. Bar graph (e): The bar chart showing the infarct volume of brains from sham-operated, peanut oil treated ischemic and C.oil post-treatment ischemic. Data are expressed as mean ± S.E.M. *P *< 0.001 was considered highly significant when comparisons were made with the peanut oil treated ischemic group by one way ANOVA followed by Newman Keuls post hoc test. Bar graph (f): Effect of post-treatment of C.oil on edema volume after 5 hrs of ischemia. Data are expressed as mean ± S.E.M. of five animals each group. *P *< 0.001 was considered significant when comparisons were made with the peanut oil treated ischemic group by one way ANOVA followed by Newman Keuls post hoc test.

After 5 hrs of ischemia in the peanut oil treatment rats the neurological scores were significantly increased (median 1.7) as compared to sham-operated rats. Post ischemic treatment with C.oil failed to offer significant reduction (median 1.3) in the neurological scores after 1 hr of treatment as compared to ischemic peanut oil treated rats (Fig. 3d).

**Figure 4:**
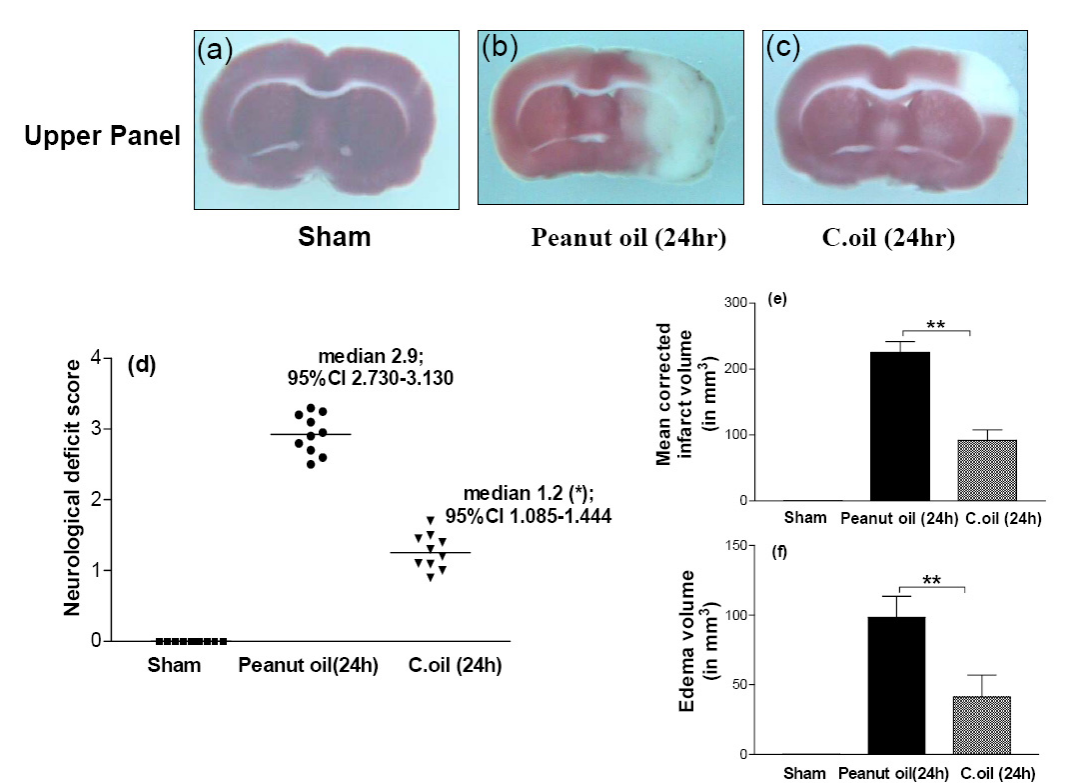
**Effect of C.oil given 4 hrs after ischemia and evaluation were made after 24 hrs of ischemia.** After 24 hrs sham-operated, peanut oil treated ischemic group and C.oil post treated group of rats were evaluated for neurological deficit thereafter rats were euthanized and infarct and edema volume were calculated. Upper panel showing the representative photograph of TTC stained coronal section from brain sham-operated (a), peanut oil treated ischemic and (b) C.oil treated after ischemia (c). Number of animals used was ten in each group. Bar graph (d): Neurological evaluation scores of rats from sham-operated, peanut oil and C.oil post-treatment ischemic groups at 24 hrs are shown in scattergram. The line demarcates the median. *P < 0.05 was considered significant when comparisons were made with the peanut oil treated ischemic group by Non parametric Kruskal-Wallis test followed by Dunn's Multiple Comparison Test. Bar graph (e): The bar chart showing the brain infarct volume after 24 hrs from sham-operated, peanut oil treated ischemic and C.oil post-treatment ischemic. Data are expressed as mean ± S.E.M. *P *< 0.001 was considered highly significant when comparisons were made with the peanut oil treated ischemic group by one way ANOVA followed by Newman Keuls post hoc test. Bar graph (f): Effect of post-treatment of C.oil on edema volume after 24 hrs of ischemia. Data are expressed as mean ± S.E.M. of ten animals each group. *P *< 0.001 was considered significant when comparisons were made with the peanut oil treated ischemic group by one way ANOVA followed by Newman Keuls post hoc test.

After 24 hrs of ischemia, the neurological scores were significantly (median 2.9) effected by ischemic peanut oil treated rats. C.oil could significant improve the neurological deficit induced by ischemia (median 1.2; P < 0.05) (Fig. 4d).

### Infarct and brain edema volume

At 5 hrs corrected infarct volume in the peanut oil treated ischemic rats (n = 5) was 148.0 ± 19.36 mm^3^. Treatment with C.oil post ischemia produced decrease in the infarct volume to 66.00 ± 14.39 mm^3^, *P *< 0.001 as compared to the peanut oil treated ischemic group (Fig. 3 Upper panel and e). Edema volume in the peanut oil treated ischemic animals at 5 hrs (n = 5) was 48.23 ± 6.36 mm^3 ^significantly increase as compared to sham-operated and C.oil post treatment. C.oil treatment decreased the edema volume to 26.36 ± 5.367.64 mm^3^, *P *< 0.001 (Fig. 3f).

At 24 hrs corrected infarct volume in the peanut oil treated ischemic rats (n = 10) was 225.32 ± 16.25 mm^3^. Treatment with C.oil post ischemia produced decrease in the infarct volume to 92.23 ± 15.69 mm^3^, *P *< 0.001 as compared to the peanut oil treated ischemic group (Fig. 4 Upper panel and e). Edema volume in the peanut oil treated ischemic animals (n = 10) was 98.36 ± 15.23 mm^3 ^significantly increase as compared to sham-operated and C.oil post treatment. C.oil treatment decreased the edema volume to 41.21 ± 15.69 mm^3^, *P *< 0.01 (Fig. 4f). None of the C.oil treated rat brain during isolation showed any signs of sub-arachnoid hemorrhage.

### Nitrate/nitrite levels

**Figure 5:**
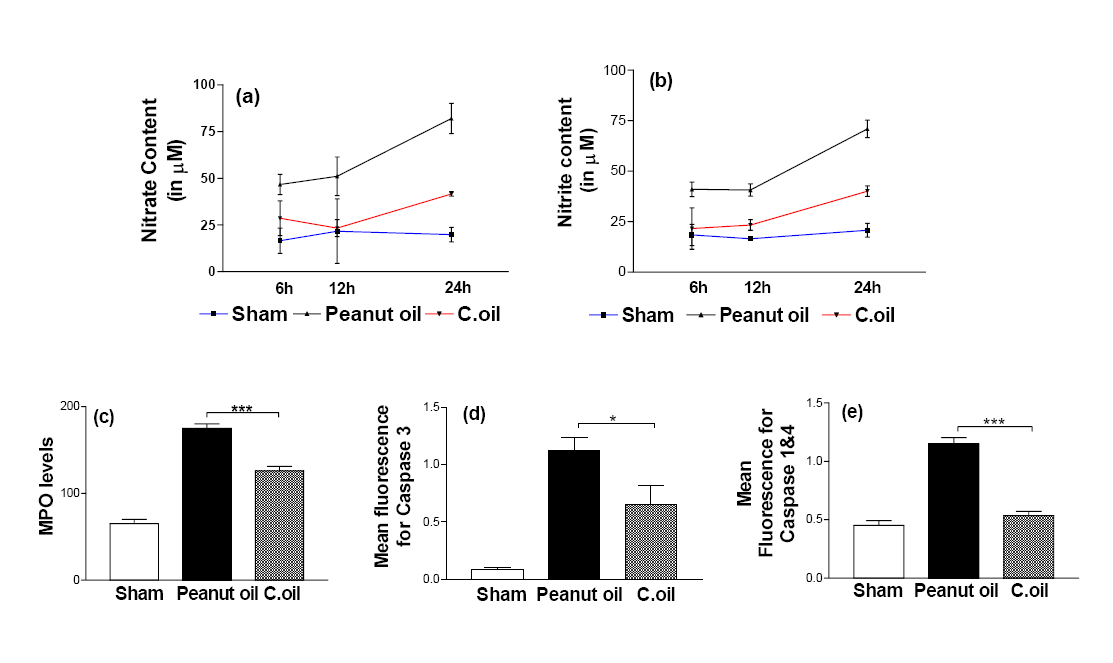
**Brain tissue Nitrate/Nitrite levels were estimated at 6, 12 and 24 hrs after ischemia.** Rats from sham-operated, peanut oil treated ischemic and C.oil treated groups were euthanized at 6, 12 and 24 hrs. At each time point five rats/group were used for the study. Data are expressed as mean ± S.E.M *P *< 0.001 was considered significant when comparisons were made with the peanut oil treated ischemic group by one way ANOVA followed by Newman Keuls post hoc test.(a): Nitrate levels and (b): Nitrite levels. (c): Effect of C.oil on myeloperoxidase (MPO) activity after 24 hrs of ischemia. Five rats per group from sham-operated, peanut oil treated ischemic and C.oil post treated were euthanized at 24 hrs Data are expressed as mean fluorescence ± S.E.M. Statistical significance was assessed by one way ANOVA. *P *< 0.001 was considered highly significant when comparisons were made between the peanut oil treated ischemic and the C.oil post-treatment group by Newman Keuls post hoc test. Neuroprotective effect of brain tissue caspase-3 (d): and caspase 1&4 (e): activity after 24 hrs of ischemia. Five rats each group from sham-operated, peanut oil treated ischemic and the treatment group with C.oil were euthanized at 24 hrs. Data are expressed as mean fluorescence ± S.E.M. Statistical significance was assessed by one way ANOVA. *P *< 0.05 was considered significant when comparisons were made among different groups by Newman Keuls post hoc test.

In the peanut oil treated ischemic rats no significant increase in the nitrate as well as nitrite level was observed at 6 and 12 hrs.(Fig. 5a and 5b) After 24 hrs post ischemia a significant increase (*P *< 0.001) in both nitrate and nitrite levels were observed in peanut oil treated rats. C.oil treatment significant decrease (P < 0.01) this ischemia induced increase in both nitrate and nitrite levels after 24 hrs post ischemia (Fig. 5a and 5b).

### Myeloperoxidase activity

The accumulation of neutrophils was investigated by measuring MPO activity from peanut oil treated animals after 24 hrs. MPO levels in the peanut oil treated ischemic brain tissue were estimated to be 175 ± 5 mU/g/min, significantly higher as compared to the sham-operated group; 65 ± 5 mU/g/min. C.oil post treatment significantly reduced the MPO activity to 126 ± 5.09 mU/g/min (*P *< 0.001) (Fig. 5c).

### Caspase-3 and caspase-1&4 enzymatic activities

Ischemia induced a significant increase in mean fluorescence obtained from the caspase-3 and caspase-1&4 activities when compared with sham-operated. C.oil treatment significantly reduced the ischemia induced increase in caspase-3 (*P *< 0.05) and caspase-1&4 (*P *< 0.001) activities (Fig. 5d and 5e).

### Flowcytometric estimations

**Figure 6:**
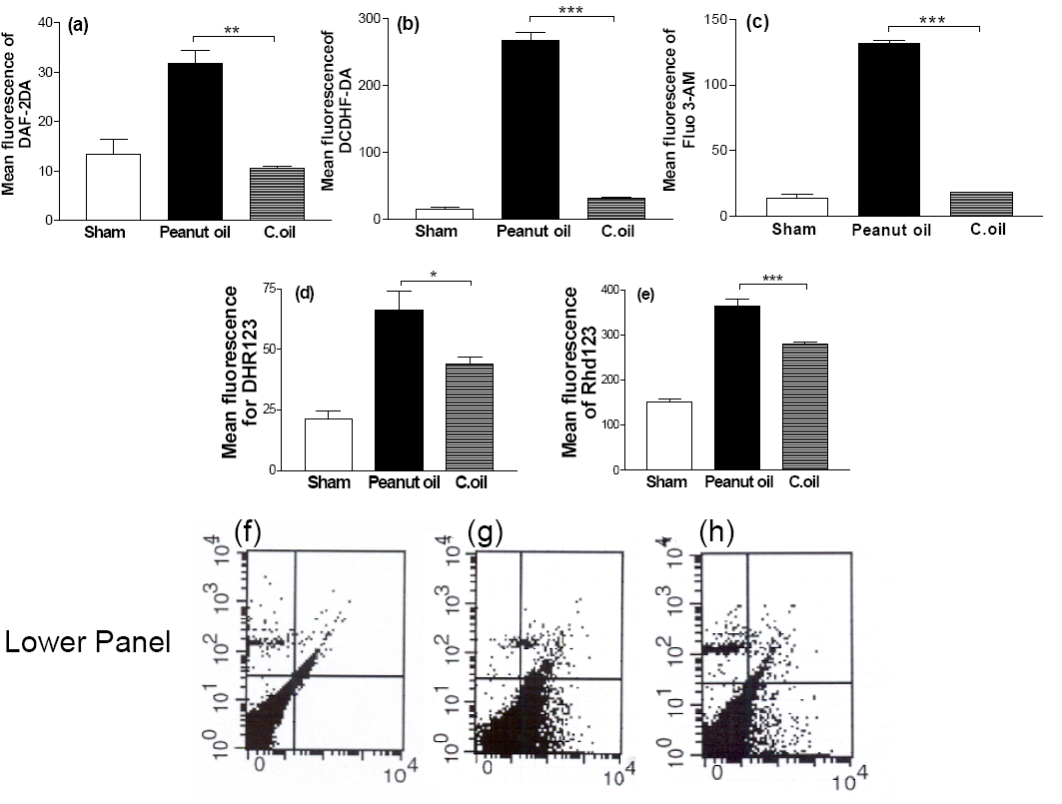
**Flowcytometric estimation of neuronal rich cell population from sham-operated, peanut oil treated ischemic and C.oil treated groups were euthanized at 24 hrs.** Brain neuronal rich cell population were loaded with different fluorochromes for the estimation of neuronal Reactive Oxygen Species (ROS), Nitric oxide (NO), Peroxynitrite (ONOO^-^), mitochondrial membrane potential (ΔΨm), intracellular calcium level and quantification of cell death type. Mean fluorescence of neurons (10^6^/ml) were acquired for quantification and analyzing in cell quest program of Becton Dickinson Flowcytometer. Data are expressed as mean fluorescence ± S.E.M *P *< 0.001 was considered highly significant when comparisons were made with the peanut oil treated ischemic group by one way ANOVA followed by Newman Keuls post hoc test. Five rats per group were used. (a): Nitric oxide from neurons (10^6^/ml) were estimated using Diaminofluoresceindiacetate (DAF-2DA). (b): ROS were estimated from neurons (10^6^/ml) using the mean fluorescence of DCDHF-DA. (c): Measurements of intracellular calcium from neurons (10^6^/ml) were estimated using, fluorescence of Fluo-3AM. (d): Peroxynitrite was estimated from neurons (10^6^/ml) using the mean fluorescence of DHR 123. (e): For estimation of mitochondrial membrane potential the mean fluorescence of Rhod 123 obtained from neurons (10^6^/ml). Lower panel: Representative dot plots for quantification of mode of death, the mean florescence, from neuron using FITC- conjugated Annexin V and Propidium iodide (PI). Neurons (10^6^/ml) were obtained from sham-operated (f), peanut oil treated ischemic (g) and C.oil post-treatment ischemic (h) groups of rats; lower left quadrant shows viable cells (Annexin V negative and PI negative), lower right quadrant shows early apoptosis (Annexin V positive and PI negative, Upper right quadrant shows late apoptotic/necrotic cells (Annexin V positive and PI positive) and upper left quadrant shows necrotic cells (Annexin V negative and PI positive).

A neuronal rich cell suspension was prepared from the forebrain of sham-operated, I/R and C.oil treated group of rat after 24 hrs of reflow. The viability of neurons was confirmed by using Apoptosis Detection kit (detailed below). Neurons were labeled with Annexin V conjugated with FITC which labels the phosphotidylserine sites on the membrane surface of the apoptotic cells and Propidium iodide (PI) to label the cellular DNA in necrotic cells. The Neuronal rich cell population from sham-operated rats was negative for both of these stains confirming that these neurons were viable (Fig. 6 lower panel f).

### Nitric oxide measurement

Significantly increased level of NO was observed in peanut oil treated group as compared to sham-operated animals. C.oil post-ischemic treatment showed significant decrease in NO content (*P *< 0.01) (Fig. 6a).

### Reactive oxygen species measurement

Rats of the peanut oil treated ischemic group showed an increased level of ROS (Mean fluorescence 266.7 ± 11.7) as compared to sham-operated animals (Mean fluorescence 15.0 ± 3). C.oil treated ischemic group showed significant decrease in ROS content (Mean fluorescence 31.1 ± 1.8) (*P *< 0.001) (Fig. 6b).

### Intracellular calcium

Isolated neurons from the brain of peanut oil treated ischemic rats after 24 hrs of clot implant showed significantly increased level of calcium as compared to sham-operated animals. C.oil treatment after 4 hrs of clot implant showed significant decreases in calcium overload. (*P *< 0.001) There was no significant difference in the calcium level of sham-operated group and C.oil treatment group (Fig. 6c).

### Peroxynitrite measurement

Significant increased level of peroxynitrite was observed in peanut oil treated ischemic group as compared to sham-operated group. C.oil treatment significantly decreases the ischemia induced peroxynitrite content (*P *< 0.05) (Fig. 6d).

### Mitochondrial membrane potential (ΔΨm)

An increase in fluorescence for **ΔΨ**m was observed in peanut oil treated ischemic group of brain neurons. The fluorescence from C.oil post treatment neurons was close to sham-operated. C.oil treatment offered significant protection as the fluorescence decreased (*P *< 0.001) (Fig. 6e).

### Neuronal death

In peanut oil treated ischemia group significantly large numbers of neurons were found apoptotic (45.37%) in comparison to the other groups. C.oil post treatment group showed reduction in cell death. The percentage of viable cells was increased significantly (80.31%), with the early apoptotic cells population reduced to 7.28% (Fig. 6 Lower panel).

### Expression of iNOS, eNOS and cytochrome c

**Figure 7:**
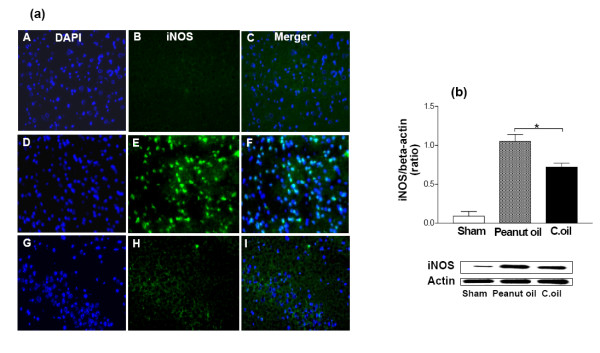
**Effect of C.oil on immunohistochemical and Western blot analysis iNOS expression after 24 hrs of ischemia in the brain.**(a): Photomicrographs (representative of 5 photomicrographs) showing inducible nitric oxide synthase (iNOS) immunoreactivity in sham-operated group (A-C); peanut oil treated ischemia group (D-F), and ischemia-C.oil post-treated groups (G-I) after 24 hrs of clot implant. Sections were immunolabeled with a polyclonal anti-iNOS antibody, which was observed as green fluorescence (B, E & H) and counterstaining by DAPI is shown as blue fluorescence (A, D & G). The merger is shown in C, F & I. Magnification: 40×. (b) iNOS expression by immunoblotting. Bar chart shows the quantification of bands. Lower Panel showing the representative bands for iNOS and β-Actin as lane 1: sham-operated, lane 2: Peanut oil treated ischemic, and lane 3: C.oil post treated (50 μg/lane). Data are expressed as mean ± S.E.M of five animals per group. (*P < 0.05) was considered highly significant when comparisons were made with the peanut oil treated ischemic group by one way ANOVA followed by Newman Keuls post hoc test.

**Figure 8:**
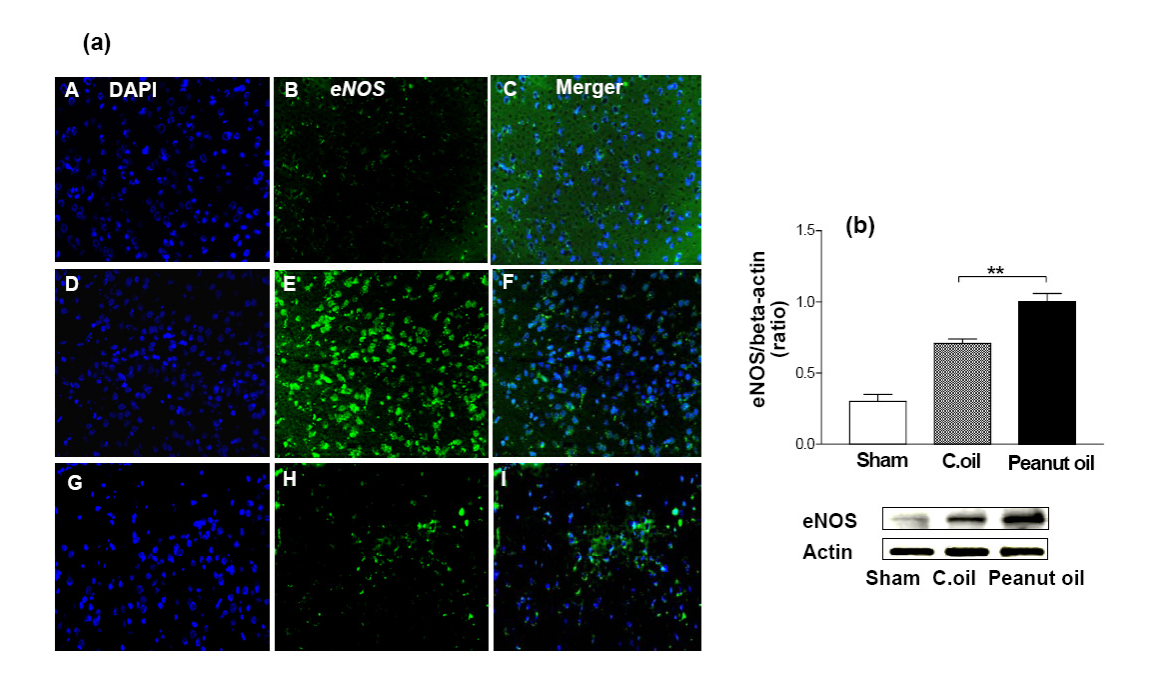
**Effect of C.oil on immunohistochemical and Western blot analysis of eNOS expression after 24 hrs of ischemia in the brain.**(a): Endothelial nitric oxide synthase immunoreactivity after 24 hrs of ischemia. Photomicrographs (representative of 5 photomicrographs) showing endothelial nitric oxide synthase (eNOS) immunoreactivity in sham-operated group (A-C); peanut oil treated ischemia group (D-F), and ischemia-C.oil post-treated groups (G-I) after 24 hrs of clot implant. Sections were immunolabeled with a polyclonal anti-eNOS antibody, which was observed as green fluorescence (B, E & H) and counterstaining by DAPI is shown as blue fluorescence (A, D & G). The merger is shown in C, F & I. Magnification: 40×. (b) eNOS expression by immunoblotting. Bar chart shows the quantification of bands. Lower Panel showing the representative bands for eNOS and β-Actin as lane 1: sham-operated, lane 2: Peanut oil treated ischemic, and lane 3: C.oil post treated (50 μg/lane). Ischemia induced eNOS expression was greatly reduced by C.oil post treatment. Data are expressed as mean ± S.E.M of five animals per group. (*P < 0.01) was considered highly significant when comparisons were made with the peanut oil treated ischemic group by one way ANOVA followed by Newman Keuls post hoc test.

**Figure 9:**
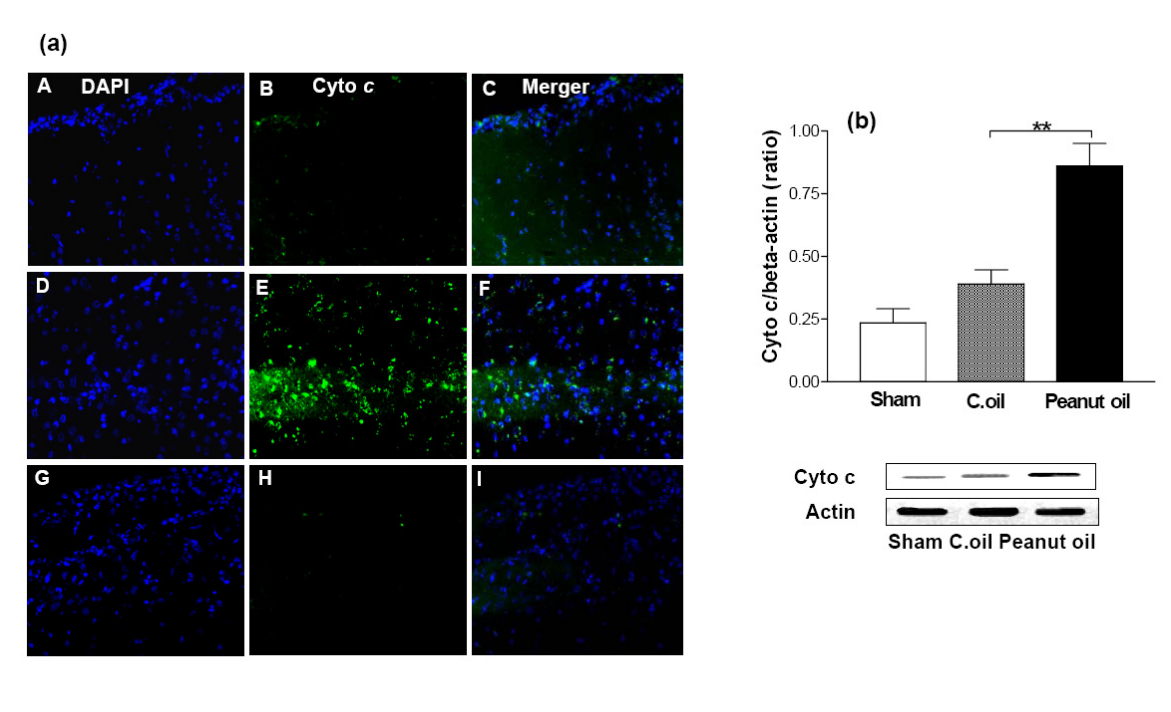
**Effect of C.oil on immunohistochemical and Western blot analysis cytochrome *c *expression after 24 hrs of ischemia in the brain. **(a): Cytochrome *c *immunoreactivity after 24 hrs of ischemia. Photomicrographs (representative of 5 photomicrographs) showing inducible nitric oxide synthase (iNOS) immunoreactivity in sham-operated group (A-C); peanut oil treated ischemic group (D-F) and C.oil post-treated groups (G-I) after 24 hrs of clot implant. Sections were immunolabeled with a polyclonal anti-cytochrome *c *antibody, which was observed as green fluorescence (B, E & H) and counterstaining by DAPI is shown as blue fluorescence (A, D & G). The merger is shown in C, F & I. Magnification: 40×. (b) Cytochrome *c *expression by immunoblotting. Bar chart shows the quantification of bands. Lower Panel showing the representative bands for cytochrome *c *and β-Actin as lane 1: sham-operated, lane 2: Peanut oil treated ischemic, and lane 3: C.oil post treated (50 μg/lane). Ischemia induced cytochrome *c *expression was greatly reduced by C.oil post treatment. Data are expressed as mean ± S.E.M of five animals per group. (*P < 0.01) was considered highly significant when comparisons were made with the peanut oil treated ischemic group by one way ANOVA followed by Newman Keuls post hoc test.

The microscope fields from midline to 2 mm lateral in the neocortex of the right and left side of each brain section were viewed. For some purposes, the usefulness of dual labeling for iNOS, eNOS and cytochrome *c *can be extended by combining it with a second fluorescent probe. For example, it can be combined with DAPI which will label the nuclei of all viable cells. Negligible immunostaining was detected for iNOS, eNOS and cytochrome *c *in the sham-operated group, whereas they were strongly expressed and some of these cell bodies were remarkably shrunken with condensed and clumped nuclear chromatin in the peanut oil treated ischemic group. Post administration of C.oil suppressed the iNOS (Fig. 7a), eNOS (Fig. 8a) and cytochrome *c *(Fig. 9a) expression after 24 hrs of ischemia. This observation indicates that post-treatment by C.oil inhibits the expression of the iNOS, eNOS and cytochrome *c*.

Confirmation of these observations was done with the help of western blot analysis C.oil post treatment significantly decreased the ischemia induced expression of iNOS (Fig. 7b) and eNOS (Fig. 8b) when compared with peanut oil treatment ischemic group after 24 hrs. The cytochrome *c *release was observed in peanut oil treated ischemic brain tissue. A significant decrease in the cytochrome c expression was observed from C.oil treated brain as compared to ischemic peanut oil treated rats (Fig. 9b).

### Expression of Bax and Bcl-2

**Figure 10:**
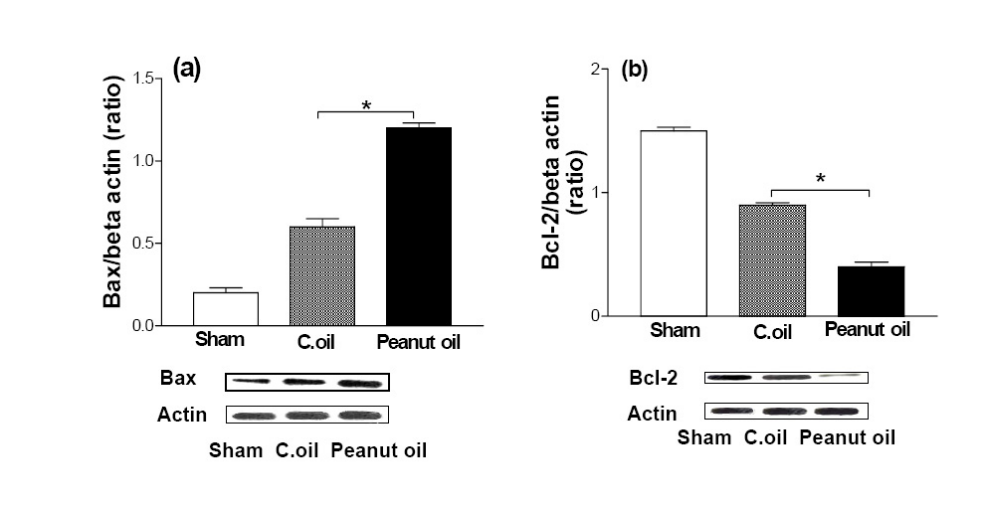
E**ffect of C.oil on Western blot analysis of Bax and Bcl-2expression after 24 hrs of ischemia in the brain.**(a) Bax expression by immunoblotting. Bar chart shows the quantification of bands. Lower Panel showing the representative bands for Bax and β-Actin as lane 1: sham-operated, lane 2: Peanut oil treated ischemic, and lane 3: C.oil post treated (50 μg/lane). Ischemia induced Bax expression was greatly reduced by C.oil post treatment. Data are expressed as mean ± S.E.M of five animals per group. *P < 0.05 was considered highly significant when comparisons were made with the peanut oil treated ischemic group by one way ANOVA followed by Newman Keuls post hoc test. (b) Bcl-2 expression by immunoblotting. Bar chart shows the quantification of bands. Lower Panel showing the representative bands for Bcl-2 and β-Actin as lane 1: sham-operated-operated, lane 2: Peanut oil treated ischemic, and lane 3: C.oil post treated (50 μg/lane). Ischemia induced Bcl-2 expression was greatly increased by C.oil post treatment. Data are expressed as mean ± S.E.M of five animals per group. (*P < 0.05) was considered highly significant when comparisons were made with the peanut oil treated ischemic group by one way ANOVA followed by Newman Keuls post hoc test.

Significant increase in the expression of Bax was observed in peanut oil treated ischemic group after 24 hrs. Expression was reduced in the C.oil post treatment group and was almost equal to sham-operated group (Fig. 10a). Significant decrease in the expression of Bcl-2 was observed in peanut oil treated ischemic group after 24 hrs. Expression was increased in the C.oil treatment group but was not equal to that in the sham-operated group (Fig. 10b).

## Discussion

The studies presented above show that C.oil significantly reduces infarct size and improves neuroscore after ischemic insult induced by MCAo rats. Moreover, C.oil treatment confers a large therapeutic window of at least 4 hours and is devoid of major hemorrhagic risk.

After an ischemic attack, brain infarction tends to spread out circumferentially from the core of the infarct and the penumbral regions undergo delayed energy-dependent apoptosis [34] and inflammation [35]. In particular, thromboembolic stroke develops in a very complicated manner; on the one hand, the ischemic lesion may enlarge over the period of time and may result in a large infarct. On the other hand, in a situation where spontaneous reperfusion can occurs, the brain tissue may be salvaged [36]. There are many reports of qualitative, but not quantitative, changes in lesion volume in thromboembolic stroke in individual rats [37]. Therefore, we designed the present study to examine how lesion volume and infarct volume after thromboembolism evolves quantitatively. We were unable to observe a significantly reduction in the early lesion (MRI). A significant attenuation was observed in volume of infarct (TTC) by C.oil treatment at 5 hrs and 24 hrs after ischemia.

Behavioral testing, in addition to histology over 5 hrs to 24 hrs after ischemia adds to the reliable evaluation of potential therapeutic interventions after embolic MCA occlusion. Assessment of functional outcome more closely reflects the approach in human clinical studies on re-canalization. Furthermore, relying only on infarct volume does not show the detection of abnormal function after ischemia in cases of normal or near-normal histology. An improvement in the neurological score was observed with C.oil treatment.

Brain edema formation, is one of the most dangerous consequences of ischemic brain injury [38]. An important factor of brain edema formation is the toxicity due to free radicals generated from lipid peroxidation. We have observed significant suppression in edema volume (as judged by hemispheric enlargement). This is an interesting finding, indicating that the anti-edematous effects of C.oil persisted for at least 24 hrs from a therapeutic point of view, this can be extremely useful. This effect of C.oil may be due to scavenging of free radicals and the prevention of lipid peroxidation as reported earlier in our report [6,14]. An important question that has not been addressed in the present study is the molecular mechanism of C.oil's anti-edema action.

Chemotactic recruitment of leukocytes starts within hours of I/R insult and these adhere to the internal lumen of blood capillaries. On the one hand, activated leukocytes produce a secondary occlusion and, on the other hand, generate a burst of significant amount of superoxide adding to the injury. The tissue MPO levels were significantly attenuated by post treatment with C.oil.

NO-induced apoptosis has been studied in a variety of cell types, and a direct action of NO on the mitochondria, leading to collapse of mitochondrial membrane potential followed by cytochrome *c *release, has been suggested as the major mechanism [39]. Accordingly, we elected to study the possible effect of NO metabolites, on the mitochondrial membrane potential and cytochrome *c *release. Cytochrome *c *release was followed by the activation of caspase-9 and -3, we next wanted to elucidate whether the pattern of activation of the caspases could be correlated to the pattern of cytochrome *c *release. There is also disagreement as to the specific role of some of these pathways [40] and the complex interactions ('cross-talk') that exist between these pathways.

In it perhaps relevant to mention in this context, that the initial rise in NO starts within 3–24 min and rises significantly from <10 nM upto1 μM during ischemia [41] due to activation of constitutive NOS. In the extra cellular milieu, NO reacts with oxygen and water to form nitrates and nitrites. In the present study, NO_*x *_levels in the ischemic brain were increased, consistent with reports from other laboratories [42] and in patients following stroke [43]. The delayed increase in NO_*x *_levels in the brain tissue after cerebral ischemia may be injurious or, at the very least, reflect injury. C.oil blunts the detrimental rise in NOx at after ischemic injury. It is more pertinent to consider the formation of peroxynitrite due to the lack of availability of NO and ROS. C.oil reduces the production of NO and ROS. In the present study, the ischemic induced decrease in functional capacity of the neuronal mitochondria was ascertained by probing the mitochondrial membrane potential (Δψm) with Rhd 123. Under the conditions of the present study, the increase in total fluorescence of Rhd 123 indicated the probable depolarization or collapse of the mitochondria within the neurons. Post treatment with C.oil prevents the increase in the fluorescence indicating a protection of the membrane potential or no depolarization of the mitochondria within the neurons. On one hand the ischemia damage to the mitochondrial respiratory chain may cause increase in mitochondrial generation of ROS and on other substantial rise in cytosolic calcium promotes the mitochondrial calcium uptake causing the collapse of (Δψm). C.oil treatment reduces the neuronal ROS and calcium rise.

We have reported recently that pretreatment by C.oil was significantly effective in limiting brain injury in an in vivo model of cerebral ischemia/reperfusion (the middle cerebral artery occlusion in rats) where the role of oxidative stress is highly implicated [6,14]. We further observed a significant rise is peroxynitrite levels as previously reported. C.oil attenuated this rise significantly. A rise in peroxynitrite levels can induce apoptosis cell death in ischemic injury [44]. If so, peroxynitrite-mediated apoptotic may also be inhibited by C.oil.

Interestingly, the resultant effect of ischemia on the neuronal calcium overload was significantly reduced by C.oil. However, it is important to mention that, in addition to interacting with calcium, C.oil has other properties that may also contribute to neuroprotection. During ischemia eNOS expression is augmented, while nNOS increases during reperfusion. It has been suggested that eNOS-derived NO may protect against ischemia by contributing to vasodilatation and by inhibiting aggregation and adherence of platelets or leukocytes [45]. It is not clearly evident whether endothelial NOS or neuronal NOS, or both caused the elevation of the NO end products seen after ischemic insults. After a transient increase in the expression of both nNOS and eNOS in the ischemic area, a secondary wave of NO overproduction starts to develop several hours after the initial ischemic insult and is sustained for up to 4–7 days [46,47] iNOS expression is much more variable in experimental stroke, as it may start around 4 hrs after MCAo [48] and 12 hrs after ischemia/reperfusion insult in transient forebrain ischemia model [49].

When iNOS expression rises, NO production naturally starts to rise. Greater NOS activity in core regions could explain in part the increased vulnerability of that region to ischemic damage and could theoretically contribute to the progression of the infarct over time [50]. Our data suggests neuroprotection shown by C.oil could primarily be due to inhibition of iNOS because we have administered the C.oil 4 hrs after initial ischemic insult. Previous studies have shown that expression of iNOS starts after 4–6 hrs after of initial ischemic insult. This observation was further substantiated by highly significant rise in NOx levels. It is well documented that expression of nNOS peaks after 1 hr of initial ischemic insult so at the time of treatment this target is already lost.

Increased expression of caspase-1, -3, -8, and -9, and of cleaved caspase-8, has been reported in the penumbra. Our results, together with the data available in literature suggest the neuroprotective effect of Caspase-1. It has the peculiarity of being involved in the activation of both apoptosis and inflammation, through the intermediation of the pro-inflammatory cytokine IL-1b [51]. Although our data demonstrated attenuation of caspase-1&4 and 3 by C.oil in transient focal cerebral ischemic injury, we do not exclude a role for cytokines in the regulation of cell death.

One of the other mechanism(s) by which the release of cytochrome *c *occurs is mediated by the Bcl-2 family, including Bax and Bid, which promote apoptosis, whereas the antiapoptotic members of the Bcl-2 family of proteins, including Bcl-2 and Bcl-xL, prevent the release of cytochrome *c *from the mitochondria [52]. Moreover, NO-induced apoptosis is related to the increase in the Bax/Bcl-xL rate, the release of cytochrome *c *and caspase activation [53]. We, therefore, studied the possible role of Bax and Bcl-2. Bax translocation to the mitochondria occurs in response to a wide variety of apoptotic stimuli and may, therefore, serve as a key interaction point for various apoptotic signals.

**Figure 11:**
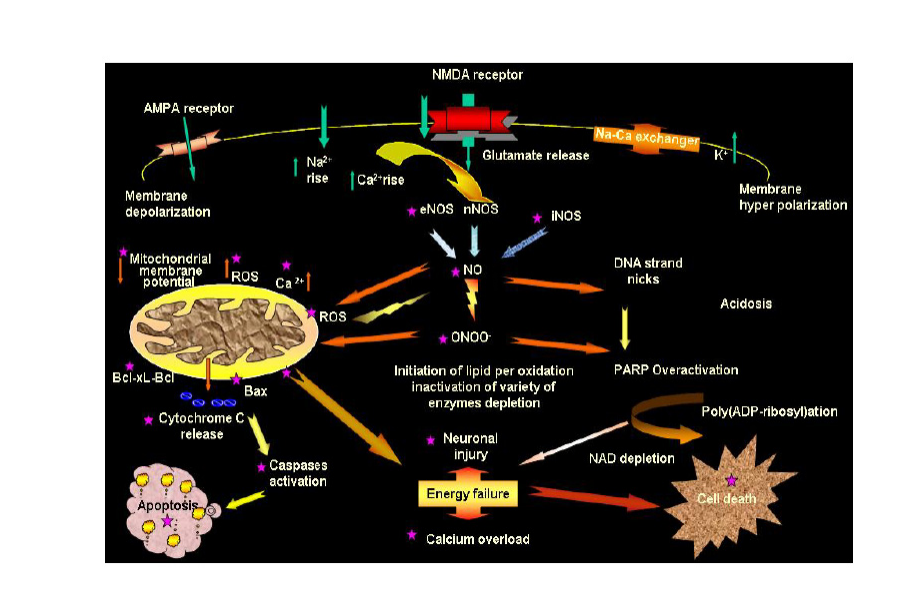
**Schematic diagram showing some of important downstream targets of apoptosis, as well as necrosis. **Neuroprotective effect of C.oil denoted by (★) effecting several targets in ischemic cascade of injury.

C.oil attenuates the up-regulation of Bax and up-regulates Bcl-2 expression; these possibly block the release of mitochondrial cytochrome *c *and prevent apoptosis. C.oil might thereby modify biochemical pathways that lead to cell death or survival. Perhaps action of C.oil in controlling both apoptosis and necrosis through up or down regulation of various pathways is advantageous as a way to ensure immediate and complete elimination of damaged cells. A complex cascade of molecular events is set in motion during cerebral ischemia and culminates in neuronal cell death some of major events are shown in Fig. 11.

## Conclusion

In brief, C.oil significantly reduces the ill effect of ischemia by attenuating nitrosative and oxidative stress. Ischemia induces collapse of mitochondrial membrane potential, cytochrome *c *release, altering the Bax: Bcl-2 ratio and subsequently caspases activation led to induction of apoptosis in sequential fashion was reverse significantly by C.oil post treatment. In conclusion, our findings provide evidence for the high efficacy of C.oil as a neuroprotective, with an excellent therapeutic window for the prevention of ischemic brain injury.

It remains an open question whether C.oil would also have neuroprotective effects in humans, but careful study in these aspects seem warranted. Recently C.oil is reported for reversing oral sub mucosal fibrosis; a precancerous condition for oral cancer, is well tolerated and has been recommended for a Phase II trial in patients [54].

## Competing interests

MR holds a patent on the applications of curcuma oil for the treatment of neurocerebrovascular disorders (please see the references quoted [14]). The other authors declare that they have no competing interests.

## Authors' contributions

PD carried out all animal care, macroscopic examination (TTC brain), immunoassays, analysis, and drafting of the manuscript. PG carried out neurobehavioral assessment, nitrate/nitrite and MPO estimations and participated in MRI experiments. US carried out MRI experiments. NRJ aided in analysis and helped in drafting the manuscript. MR conceived of the study, participated in the design of the experiment and in coordination and the writing of the manuscript. All authors have read and have approved the final manuscript.

## Pre-publication history

The pre-publication history for this paper can be accessed here:


